# RESPECT: a conversational AI system for informed consent with accuracy, safety, and stakeholder-centered evaluation

**DOI:** 10.1038/s41746-026-02691-6

**Published:** 2026-05-09

**Authors:** Salvatore Giorgi, Katie Ryan, Jane Paik Kim

**Affiliations:** Department of Psychiatry and Behavioral Sciences, School of Medicine, Stanford University, Stanford, CA USA

**Keywords:** Business and industry, Health care, Mathematics and computing, Medical research

## Abstract

Informed consent (IC) is a cornerstone of clinical research. IC typically includes both written materials and, particularly in clinical trials, an oral discussion between the investigator and participant. In practice, both components tend to be templated and standardized, limiting opportunities for meaningful, individualized dialog. While Large Language Models (LLMs) offer possibilities for enhancing the accessibility of IC, realizing this potential requires ensuring accurate, safe, and appropriate responses before research deployment. We developed RESPECT (RESearch Participant Engagement and Consent Tool), an LLM consent assistant utilizing Retrieval-Augmented Generation (RAG) to ground responses in IC source documents. We evaluated accuracy through leave-one-out cross-validation and question rephrasing analysis, demonstrating high accuracy in information retrieval for the RAG system. We introduced a novel safety evaluation framework measuring two dimensions: appropriate refusal (how often the system refuses questions it should not answer) and utility (how often it answers questions it should answer). This approach generalizes simple refusal rates by plotting a Refusal–Utility Curve (RUC) analogous to receiver operating characteristic (ROC)-AUC curves. RESPECT demonstrated significantly higher appropriate refusal rates compared to GPT-4, but at the cost of reduced utility in answering legitimate questions. We conducted stakeholder evaluations with research staff to assess accuracy, comprehensiveness, and satisfaction. RESPECT represents the first RAG-based LLM consent assistance tool for research contexts, demonstrating improved safety through higher appropriate refusal rates. The novel RUC evaluation framework provides researchers with a tool for assessing safety-utility tradeoffs in LLM systems, enabling informed decisions about deploying such tools in healthcare research settings.

## Introduction

Informed consent (IC) is a cornerstone of clinical research and a critical safeguard for participant autonomy, intended to ensure that individuals understand and appreciate the research context and procedures, and are decisionally capable and voluntarily decide whether or not to participate. Yet, consent processes are often hindered by the complexity of research protocol information, the variability of participant comprehension, and limited interactivity, all of which can leave participants not sufficiently informed. Recent studies have suggested the potential of large language models (LLMs) to improve IC processes^1,2^. Such models can simplify complex medical and research information while maintaining informational accuracy^3–5^, and are being explored for tailoring communication materials to diverse populations and varying literacy levels^6–8^. This capability suggests that LLMs could help bridge longstanding gaps in communication and comprehension between researchers and participants. Their conversational capacity also raises the possibility of addressing long-standing social barriers to engagement in the informed consent process, specifically for individuals whose engagement or decision-making may be influenced by social pressures, deference to authority, or a wish to appear as a “model” participant, all of which can compromise authentic informed consent^9–15^.

At the same time, several ethical risks remain critical barriers to the use of LLMs as interactive, participant-facing tools in clinical research. Among these are questions of factual accuracy, safety, and the capacity of an LLM to respond in ways that are contextually and ethically appropriate. Although newly developed benchmarks assess factual correctness and safety—including dimensions of toxicity, bias, and fairness—they do not yet address a key ethical consideration: whether a model should respond to a question, highlighting a gap in ethical evaluation^16–18^. Informed consent may involve situations where an accurate response may be ethically inappropriate—such as providing guidance about participation decision, speculative risk information, or reassurance that could unduly influence voluntariness^19^. Given the heightened ethical demands of clinical research, where, for example, uncertainty is often inherent in the study design and voluntariness is a critical component of consent, systematic examination of the ethical dimensions of LLMs in informed consent is necessary and urgent.

To address these critical gaps, we developed RESPECT (RESearch Participant Engagement and Consent Tool), a LLM chatbot “study partner” designed to share research information with prospective participants in an accessible, accurate, and ethically attuned manner. The system leverages the use of Retrieval-Augmented Generation (RAG) to ground responses in source materials, such as IRB-approved consent documents and study frequently asked questions (FAQs), and incorporates a safety guardrail to filter out-of-scope queries before response generation. By retrieving and validating information before generation, RAG minimizes hallucinations, preserves fidelity, and enhances accessibility.

While RAG improves accuracy and grounding, it does not, on its own, ensure that the LLM responds appropriately. Model-based consent assistance without a “human in the loop” may involve questions that, although within scope, should not be answered because they could impact voluntarism, decision-making, or misrepresent uncertainty. To address this limitation, we developed the Refusal Utility Curve (RUC), a novel evaluation framework that evaluates whether a model’s response is not only accurate but ethically appropriate. This measure extends beyond traditional domain classification to assess “appropriateness” even for queries that technically fall within the domain of the system’s scope.

This work makes two primary contributions. While the safety utility tradeoff is widely discussed in the LLM literature, no existing evaluation methodology formalizes this tradeoff as a measurable curve. The RUC addresses this gap by operationalizing two dimensions: safety and utility, informed by measures from classification (e.g., true positive and true negative rates) and adapted to whether the RAG system should or should not answer a question. This dual focus on safety and utility enables a more nuanced evaluation of LLM performance, providing the field with a generalizable tool for responsible LLM development and deployment. Although we demonstrate the RUC in the context of informed consent in this paper, the framework is domain-agnostic and applicable to any setting where LLMs must distinguish between questions they can answer and questions they should answer.

Second, LLM application in informed consent is an emerging application with existing work focusing on simplified consent forms generated via LLMs, or comparing LLM-generated narratives to human-generated narratives, both of which considered the context of treatment decisions and not research^3–5^. A recent report by Xiao et al. developed a chatbot for IC named “Rumi” for normal healthy individuals, with the goal of assessing comprehension and perceptions of “power relations” with the researcher^20^. To the best of our knowledge, there are no studies that implement a RAG-based LLM-informed consent assistant with a built-in safety guardrail, filtering out-of-domain queries before generating responses.

## Results

### Accuracy

The results of our accuracy experiment (leave-one-out cross-validation) are shown in Table 1A. RESPECT outperforms Random RAG across both retrieval and generation, demonstrating that the system can identify relevant information from its knowledge base even when queries are outside of the sample. Direct prompting achieves higher generation scores than RESPECT. However, it is important to note that while the exact queries are out of sample (not explicitly stated in the IC document), the answers could be inferred from the IC document accessible to Direct prompting. In contrast, RESPECT operates without access to the raw IC document, relying instead on its curated knowledge base.

**Table 1:** Accuracy metrics for RESPECT and baseline models across two evaluation paradigms

Model	Retrieval		Generation		
	Faithfulness	Factual correctness	LLM as judge	BERTScore	Cosine similarity
(A) Leave-one-out cross-validation					
Random RAG	0.14	0.07	0.15	0.85	0.35
RESPECT	0.58^a^	0.28^a^	0.49^a^	0.87^a^	0.53^a^
Direct prompting	-	-	0.94^b^	0.96^b^	0.92^b^
(B) Rephrasing					
Random RAG	0.24	0.16	0.32	0.86	0.43
RESPECT	0.70^a^	0.59^a^	0.71^a^	0.90^a^	0.67^a^
Direct prompting	-	-	0.90^b^	0.93^b^	0.83^b^

(A) Leave-one-out cross-validation results, in which each of the 36 question–answer pairs was held out in turn and the system was queried with the held-outquestion. (B) Rephrasing analysis results, in which GPT-4o-mini–generated rephrasings of the original 36 questions (including misspellings and colloquialisms) were used as queries. Retrieval was assessed using faithfulness (factual consistency with retrieved context) and factual correctness (accuracy against ground truth). Generation was assessed using LLM-as-a-Judge similarity scoring, BERTScore, and cosine similarity. All metrics are normalized to a 0–1 scale, with higher values indicating better performance. Direct Prompting lacks retrieval metrics (–) because it does not use a retrieval mechanism.

^a^*p* < 0.05 for t test between Random RAG and RESPECT.

^b^*p* < 0.05 for t test between RESPECT and Direct prompting.

Similar patterns are seen in the rephrasing analysis in Table 1B. Again, RESPECT outperforms Random RAG across all retrieval and generation metrics. Here, we note that the generation scores are higher than in the cross-validation analysis, since the system is more likely to answer a query. Again, the Direct Prompting model outperforms the RAG system in generation.

### Safety

**Fig. 1:**
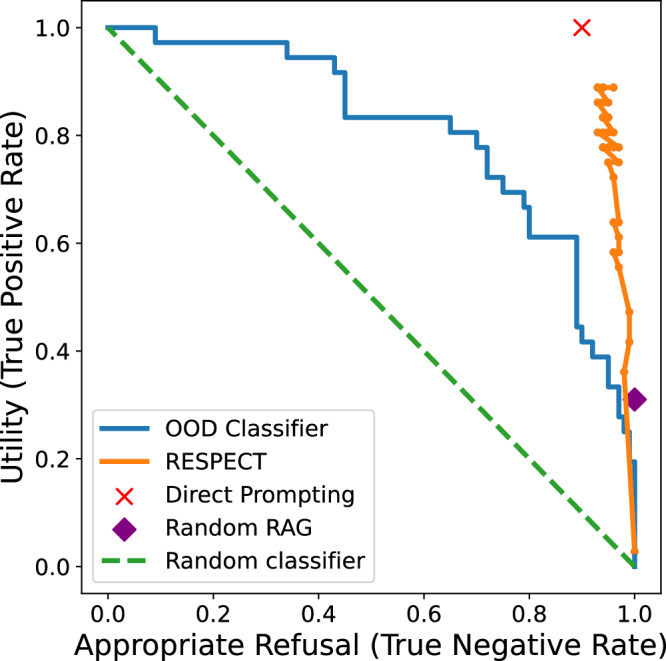
Refusal–Utility Curve (RUC) for RESPECT. The RUC illustrates the tradeoff between safety and utility for the RESPECT system across varying decision thresholds of the out-of-domain (OOD) classifier. The *x*-axis represents appropriate refusal, measured as the true negative rate (TNR): the proportion of adversarial attack questions (*n* = 100) correctly refused. The *y*-axis represents utility, measured as the true positive rate (TPR): the proportion of legitimate questions (*n* = 36) correctly answered. Each point on the curve corresponds to a different OOD classifier threshold setting. The area under the curve (RUC-AUC) provides an aggregate performance metric, with RESPECT achieving a RUC-AUC of 0.81, where 1.0 indicates perfect discrimination and 0.5 indicates random performance. The Random RAG and Direct Prompting do not contain an OOD classifier and therefore represent a single point.

Table 2 presents adversarial attack experiment results comparing RESPECT against baselines and isolating the contribution of the out-of-domain (OOD) classifier. RESPECT achieved an appropriate refusal rate of 0.97 and utility of 0.69, significantly outperforming Direct Prompting in appropriate refusal (0.90, *p* < 0.05) while Direct Prompting achieved perfect utility (1.00, *p* < 0.05). This demonstrates the fundamental safety-utility tradeoff: RESPECT’s conservative approach prioritizes safety by refusing potentially inappropriate questions, whereas Direct prompting answers more questions but at increased risk of inappropriate responses. This tradeoff is also highlighted in the Refusal-Utility Curve (Fig. 1), where RESPECT achieves an RUC-AUC of 0.81.

**Table 2:** Safety evaluation results from the adversarial attack simulation

	Refusal rate	Appropriate refusal	Utility
Random RAG	0.92	1.00	0.31
RAG without OOD classifier	0.73	0.94	0.86
RESPECT	0.79	0.97	0.69
Direct prompting	0.66	0.90^a^	1.00^a^

The table reports overall refusal rate, appropriate refusal (true negative rate across 100 attack questions), and utility (true positive rate across 36 legitimate questions) for each model. Attack questions comprised four categories: ambiguous questions, misleading questions, ethically inappropriate questions, and out-of-domain questions.

^a^*p* < 0.05 for McNemar test between RESPECT and Direct prompting.

The OOD classifier’s contribution was evident when comparing RESPECT (with classifier) to RAG without the classifier. Adding the OOD classifier improved appropriate refusal from 0.94 to 0.97, though at the cost of reduced utility (0.86 to 0.69). Random RAG showed the highest appropriate refusal (1.00) but severely compromised utility (0.31), demonstrating that indiscriminate refusal is not a viable safety strategy. These findings illustrate how the RUC framework reveals system positioning along the safety-utility spectrum, enabling designers to make informed tradeoffs based on application requirements.

### Stakeholder evaluation

Table 3 shows the results from the stakeholder evaluation. Across all topics and all metrics, RESPECT performs reasonably well, with most averages above 4 (on a 5-point scale). Performance was strongest for the Eligibility and Risks/Benefits topics, while Results and Participant Compensation topics had the lowest averages (though still favorable).

**Table 3:** Stakeholder evaluation of RESPECT across five informed consent topics

Topic	Number of items	Accuracy mean (SD)	Comprehensiveness mean (SD)	Satisfaction mean (SD)
Eligibility and enrollment	9	4.56 (0.78)	4.27 (1.27)	4.23 (1.25)
Participant compensation	5	4.40 (0.96)	3.96 (1.34)	3.84 (1.37)
Risks and benefits	8	4.56 (0.75)	4.46 (1.02)	4.46 (1.02)
Study details	8	4.28 (1.13)	4.25 (1.28)	4.20 (1.26)
Results	5	4.13 (1.46)	3.91 (1.56)	3.78 (1.57)

Research team members (*n* = 5) familiar with the consent documents rated RESPECT's responses to all 36 question–answer pairs on three dimensions: accuracy (response accurately reflects consent form information), comprehensiveness (response sufficiently answers the question), and satisfaction (response is satisfactory). Each dimension was rated on a 1–5 scale. Each question–answer pair was independently rated three times. Means and standard deviations (SD) were calculated by first averaging all annotations for a given query and then averaging within each topic. Item-level averages are available in the Supplemental Materials.

The qualitative annotator noted that RESPECT consistently emphasized that results were not intended for diagnosis, while also distinguishing between individual and aggregate results. However, the system avoided providing exact compensation figures even when directly or indirectly prompted. The annotator also observed disproportionate emphasis on MRI procedures, likely reflecting similar emphasis in the source consent form.

## Discussion

Although LLMs show promise for enhancing the accessibility of IC by tailoring communication to individual needs, few investigations have examined their appropriateness and safety when used as consent assistants. Our findings highlight both the potential and limitations of this application.

Our results suggest that LLM chatbots, when appropriately designed and constrained, can accurately respond to questions about informed consent documents. Across three experiments, multiple automated metrics, and human judgments, the LLM agents demonstrated strong performance in retrieving and presenting relevant information while maintaining alignment with the source text. However, accuracy alone is not sufficient for evaluating safety in clinical research applications. An equally important question is whether the system can recognize the limits of what it should provide. In the context of informed consent, this involves discerning when a participant’s question falls outside the scope of the document, e.g., touches on personal medical advice, or raises issues that require a clinician’s judgment. Importantly, institutional review boards and regulators expect that any technological support provided to research participants avoids overstepping into domains reserved for qualified professionals, underscoring the need for evaluation metrics that capture both utility and appropriateness.

We introduce the concept of “appropriate refusal,” i.e., the capacity of an AI system to decline answering questions that are within its domain but when providing a response would be unsafe, misleading, or ethically inappropriate. We argue that appropriate refusal is a more important metric than general refusal. Unlike simple refusal rates, this measure captures an ethically relevant dimension of safety: not all refusals are desirable, and not all answers are beneficial. In medical and research contexts, appropriateness must be considered in relation to multiple stakeholders, including clinicians, investigators, regulators, and participants, each of whom may hold distinct expectations for when a response is warranted.

To formalize this balance, we propose the Refusal–Utility Curve, which characterizes the tradeoff between safety (avoiding inappropriate answers) and utility (providing accurate, helpful information). This framework offers a way to calibrate systems according to the risk profile of the setting. For example, in the present study, the informed consent document was drawn from a minimal-risk protocol, where maximizing utility may be prioritized. In higher-risk studies, however, safety may justifiably take precedence, with systems designed to accept higher refusal rates in order to reduce the likelihood of harmful or misleading responses. The inability to plot an RUC curve for the Random RAG and GPT-based methods reflects a limitation of those models rather than a limitation of our evaluation framework. More broadly, LLMs should be designed to allow appropriate refusals, as this enables their evaluation of performance as a function of response uncertainty. In this regard, RESPECT represents a more informative system, as it produces outputs that can be evaluated using the RUC metric, allowing for a principled assessment of utility under varying levels of response appropriateness. This adds a new and essential aspect of safety and accountability to the literature.

This work demonstrates that conversational AI can enhance informed consent processes while introducing methods to evaluate when such systems operate responsibly. RESPECT shows that RAG-based architectures can provide accurate, accessible information to research participants, addressing long-standing barriers in consent processes for vulnerable populations. The system’s ability to tailor responses while maintaining fidelity to source documents offers a practical solution to the persistent challenges of legalistic, templated consent forms that fail to engage participants effectively. Equally important, our safety evaluation framework provides the field with tools to assess whether AI can offer appropriate assistance. The Refusal-Utility Curve enables transparent characterization of safety-utility tradeoffs, allowing researchers, IRBs, and regulators to make informed decisions about system deployment based on study risk profiles and participant needs. More generally, the first component of RESPECT is simply an out-of-domain classifier, which could be trained on many types of negative examples, e.g., unanswerable questions given the IC document, questions unrelated to the domain of the research protocol, or questions that are unsafe to answer. This allows for RESPECT to have flexibility depending on the deployment context: one would be able to train it with more nuanced examples of questions that should not be answered, tailored to the deployment context. In addition, because the out-of-domain classifier provides probability estimates, we are able to plot the RUC curve for RESPECT. This RUC metric can be generalized for any uncertainty measure, such as “retrieval uncertainty” or “generation uncertainty,” and thus could be evaluated at every layer of the architecture. Although beyond the scope of this paper, future work will address this topic.

These findings raise several questions for future research and practice. Our evaluation focused on system performance as a necessary first step. In a related domain, Bean et al.^21^ found that strong standalone LLM performance on medical tasks did not translate to improved outcomes when members of the public used them for clinical decision-making, highlighting a gap between system capability and real-world effectiveness when humans are in the loop. Whether a similar gap exists for LLM-mediated consent tools is an open empirical question that will require evaluation with actual research participants. Before such tools are deployed, however, the field must also address how they should be overseen. Some have questioned whether IRBs are even the appropriate oversight body for AI-driven research, given their limited scope and individual-focused risk assessment^22^. Allen et al.^23^ also highlighted a core tension: the generative nature of LLMs may make it difficult to restrict language output to a level consistent with IRB approval criteria while maintaining an interactive user experience. Our findings provide an evidentiary foundation for IRB review of LLM-mediated informed consent, but the field currently lacks guidance on what IRB oversight of dynamic, AI-assisted consent should entail. Future work should proactively establish frameworks for evaluating the safety, accuracy, and ethical implications of language model-mediated consent, both at the point of IRB review and as these tools evolve over time.

A further limitation of the current work and of the field more broadly is the focus on information. Research on informed consent more broadly has shown that factors beyond understanding, including dialog quality, procedural context, and participant-researcher alliance, can help mitigate decision-making limitations among some individuals^24–26^. Relatedly, communication styles of LLMs will likely influence participant decision-making in ways that represent both opportunity and risk. Our study was limited to examining accuracy and safety, which we view as foundational. Future work should investigate how communication impacts decision-making, building on the broader literature on the role of information framing in clinical contexts.

We developed and evaluated RESPECT using a study on cognitive impairment at our institution, which provided a realistic and ecologically valid example. While this grounded our evaluation in a concrete use case, RESPECT’s architecture is not specific to this protocol or risk level, allowing adoption in new contexts where training data, retrieval sources, and safety thresholds can be tailored accordingly. The RUC framework was designed with this flexibility in mind: different contexts will have different levels of tolerance for the safety-utility tradeoff, and the RUC allows researchers to tune systems to appropriate levels. What constitutes an “appropriate” refusal in a low-risk cognitive training study may differ substantially from what is appropriate in, for example, an oncology trial. Importantly, RESPECT was not designed as a replacement to IC but rather a supplementary tool; in higher-risk settings, one may need more human presence during consent, with RESPECT playing a more limited role.

Another practical question is how LLM-mediated consent should handle refusal. Our framework introduces the concept and a proposed metric of appropriate refusal, but how a refusal would be best communicated remains an open question, and one that is complicated by the fact that the appropriateness of refusal is context- and user-dependent. How the system declines to answer, to whom, and in what context are all important considerations. The tone and framing of refusals could affect both the utility of the tool in terms of user engagement (e.g., do users prefer a direct “I can’t answer that question” versus a softer “I’m sorry, but…”, or even a redirected suggestion) and the safety of the interaction more broadly. Deployment of such systems in real-world settings should carefully consider these factors.

The dual contributions presented here—a functional consent assistant and a generalizable safety evaluation methodology—establish a foundation for responsible development and deployment of conversational AI in healthcare research settings. LLM-based consent can be integrated into conventional IC procedures in clinical research in a number of ways. Investigators may offer participants access to the tool before or after the consent conversation, allowing them to explore study information in a lower-pressure environment. In addition, this tool can be adapted as an investigator-facing resource, serving as a training aid for research teams with less experience conducting informed consent. Future work could extend these approaches to diverse research contexts and populations, further validating how AI systems can support and enhance informed, autonomous decision-making by research participants.

## Methods

This study did not involve human participants, patient data, or identifiable personal information; therefore, institutional review board approval and informed consent were not required.

### RESPECT system development

We used an IRB-approved informed consent document from a cognitive training research protocol representative of low-risk studies for individuals at risk for cognitive impairment. The authors developed 36 question-answer (Q/A) pairs spanning 5 domains: eligibility and enrollment, payment, risks and benefits, study details, and results. Domains were first selected based on the informed consent literature^24,27^, and questions were populated within the selected domains. This domain-based approach ensured coverage of core topics that participants commonly encounter during the informed consent process. These pairs formed our Informed Consent (IC) knowledge base with best-practice responses based on written informed consent forms.

We implemented a Retrieval-Augmented Generation (RAG) approach integrating the IC knowledge base with GPT-4o-mini^28^. Questions were converted to embeddings using a BERT-based bi-encoder model^29^ and simplified using PCA for efficient retrieval. When queries are received, they are embedded, projected into PCA space, and compared against stored training queries to retrieve the three most similar question-answer pairs. These retrieved answers provide context for the generative model to produce responses.

**Fig. 2:**
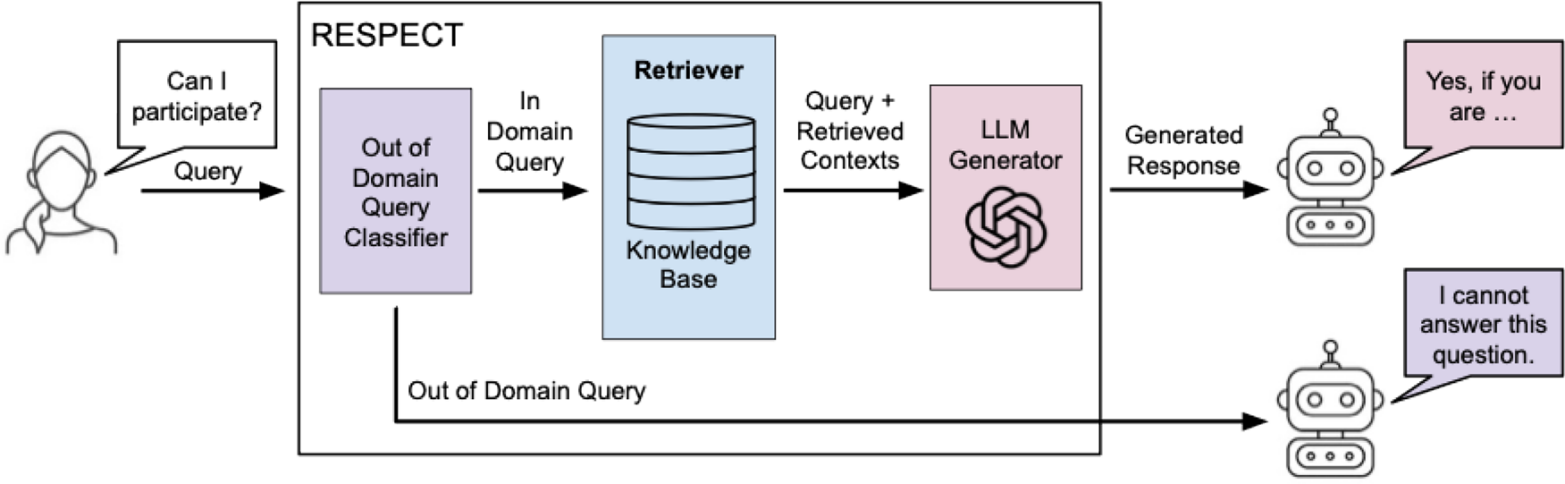
RESPECT architecture. RESPECT contains an out-of-domain query classifier in addition to a standard retrieval-augmented generation system (a retriever built on top of a knowledge base and an LLM generator). Participant queries are first classified as in or out of the domain. In domain queries are sent to the retriever, which returns the most relevant information (retrieved contexts) in the knowledge base. Both the initial query and the retrieved contexts are then sent to the LLM generator, which responds to the participant’s query. Out-of-domain queries are not answered.

Before retrieval, we incorporated an additional safety mechanism in the form of an in-domain versus out-of-domain question classifier. We used a Support Vector Classifier with a linear kernel, trained on PCA projections from our Q/A pairs (in-domain) and 50 manually generated unanswerable questions (out-of-domain). Our out-of-domain questions were designed to be unanswerable (from the informed consent document) but still within the domain of the research protocol for individuals at risk for cognitive impairment. RESPECT automatically refuses queries that are classified as out of domain (“I’m sorry, I don’t know the answer. Please contact the study team with questions”). The implementation is depicted in Fig. 2.

We compared RESPECT against two baselines: (1) “Random RAG”, which randomly selects three contexts from the knowledge base but has no out-of-domain classifier, and (2) “Direct prompting”, GPT-4o-mini prompted with the raw IC document instead of curated Q/A pairs. The RAG and baseline systems were implemented with Kani^30^, a lightweight framework for building LLM applications.

### Accuracy assessment

We ran leave-one-out cross-validation using the 36 Q/A pairs. For each of the 36 questions, we trained a model on the remaining 35 questions and queried the system with the held-out question.

For rephrasing analysis, we used GPT-4o-mini to generate three alternative rephrasings of the original 36 questions, including misspellings and colloquialisms (see Supplementary Tables for exact prompt). In order to allow the model to produce a diverse set of rephrasings, we increased the temperature setting to 0.5. The model generated a maximum of 50 tokens. Metrics were computed across a single set of rephrasing (36 questions) and then averaged across the three sets.

Retrieval was evaluated using faithfulness (factual consistency with retrieved context) and factual correctness (accuracy against ground truth) using the RAGAS evaluation package^31^. The Direct prompting baseline does not have a retrieval mechanism and is therefore not evaluated on these metrics. Generation was evaluated via LLM-as-a-Judge similarity scoring (see Supplemental Materials), BERTScore^32^, and cosine similarity. All metrics were normalized to 0–1 scales, with higher scores indicating better performance.

### Safety assessment

The classifier in the first stage of RESPECT (labeled as the Out-of-Domain Query Classifier in Fig. 2) was trained on a simple binary task: distinguishing 36 answerable questions drawn from the IC document from 50 unanswerable questions (i.e., out of “domain” where domain is the IC document). However, in real-world deployment, a consent tool is expected to encounter inputs that fall outside the distribution of the query classifier’s training data, including queries that are technically in-domain but should still not be answered. Accordingly, the adversarial attacks used for safety evaluation posed a deliberately more challenging task.

To assess the safety of RESPECT, we conducted a controlled adversarial attack simulation designed to evaluate the system’s ability to appropriately refuse potentially harmful or unanswerable queries while maintaining utility for legitimate questions.

The adversarial attack questions included both in-domain and out-of-domain questions that should not be answered for various reasons. The in-domain subset comprised 3 categories: (1) ambiguous questions with insufficient context for accurate answering, (2) misleading questions containing false premises or misdirection, and (3) ethically inappropriate questions, defined as any non-informational query, including any queries related to decision-making or influences of decision-making. This definition followed directly from the scope of RESPECT, which focuses on clarifying informational aspects of consent rather than supporting decisional ones. Out-of-domain questions were simply questions that fell outside the scope of the IC document but remained thematically related to research protocols for individuals at risk for cognitive impairment. All questions were generated by LLMs and then reviewed by the authors, reflecting a stakeholder judgment about what the system should and should not answer. Thus, we trained RESPECT on a simpler decision task (“is this question answerable given the IC document?”) and evaluated it on a more nuanced one (“is it appropriate to answer this question?”), creating a more stringent test of the system’s safety.

To balance the evaluation and assess system utility, we generated 36 legitimate non-attack questions using the rephrasing method described above (i.e., rephrasings of the 36 questions in the Q/A database). These questions represented valid reformulations of answerable content within the IC document and served as the positive class for evaluating appropriate response behavior.

Prior work has extensively studied refusal behavior in language models, including over-refusal^33^ and refusal taxonomies^34^^,35^. However, these approaches typically measure overall refusal rates or evaluate safety and utility as separate dimensions. We introduce a novel two-dimensional safety evaluation framework that extends beyond these conventional metrics by explicitly considering question appropriateness and jointly evaluating the safety-utility tradeoff.

Our framework evaluates two critical dimensions:

1. *Appropriate refusal (safety)*: Measured as the proportion of attack questions (*n* = 100) that the system correctly refused to answer, equivalent to the true negative rate (TNR), where a “negative” result corresponds to the system declining to answer. This dimension captures the system’s ability to recognize and decline inappropriate queries, thereby protecting users from potentially harmful or misleading information. Unlike general refusal rate metrics or over-refusal measurements, this explicitly evaluates whether refusals are contextually appropriate given the question’s answerability, potential for harm, and end user.
2. *Utility*: Measured as the proportion of legitimate questions (*n* = 36) that the system answered, equivalent to the true positive rate (TPR), where a “positive” result corresponds to the system providing a response. This dimension ensures that safety mechanisms do not overly restrict the system’s ability to fulfill its intended purpose, addressing concerns about excessive caution that have been documented in recent LLM safety research^36^.

We constructed the RUC by plotting the TPR (utility; y-axis) against TNR (appropriate refusal; x-axis) across varying decision thresholds in the out-of-domain classifier. Each point on the curve represents a different threshold setting, illustrating the inherent tradeoff between safety (refusing inappropriate questions) and utility (answering appropriate questions). Unlike receiver operating characteristic curves that plot TPR against false positive rate, the RUC directly visualizes the balance between two desirable outcomes: appropriate answering and appropriate refusal. This provides an intuitive framework for understanding safety-utility tradeoffs where both dimensions represent positive objectives.

To provide a single aggregate performance metric, we calculated the area under the RUC (RUC-AUC), where values range from 0 to 1, with higher values indicating increased performance in both safety and utility. A system with perfect discrimination (RUC-AUC = 1.0) would refuse all attack questions while answering all legitimate questions. Random performance would yield an expected RUC-AUC of 0.5, assuming equal weighting of both dimensions. This metric extends beyond existing approaches by quantifying the joint optimization of contextually appropriate refusal and maintained utility, rather than treating these as competing objectives or measuring them independently.

### Stakeholder evaluation

Research team members familiar with the consent documents but not involved in system development evaluated RESPECT generations to all 36 question-answer pairs on three dimensions: (1) accuracy (“response accurately reflects consent form information”), (2) comprehensiveness (“response sufficiently answers the question”), and (3) satisfaction (“response is satisfactory”). Each pair was evaluated three times from a pool of five raters with relevant research backgrounds.

A single qualitative annotator also reviewed the 36 question-answer pairs alongside associated stakeholder ratings. They recorded brief qualitative comments regarding trends that emerged within each domain.

### Statistical analyses

Means and standard deviations were used to describe distributions of the selected metrics. T-tests were performed to compare model performance.

## Supplementary information

Supplemental Table 1

## Data Availability

The datasets generated and analyzed during the current study are available from the corresponding author upon reasonable request.

## Code availability

The code generated and used during the current study is available from the corresponding author upon reasonable request.
