# Supplementary material for: RESPECT: a conversational AI system for informed consent with accuracy, safety, and stakeholder-centered evaluation
Source: NPJ Digit Med. 2026 May 9;9:552. doi: 10.1038/s41746-026-02691-6 (PMC13377031; doi:10.1038/s41746-026-02691-6)
Supplement: Supplementary file 1 — Supplemental Table 1 [file 41746_2026_2691_MOESM1_ESM.pdf]

Supplemental Materials

Sample Queries and Responses

Supplemental Table 1. Sample of queries and responses by topic.

Topic	Prompt	RAG Response
**Eligibility and Enrollment**	how do I go about getting myself signed up for this? like, is there a specific process or steps i need to follow? I'm kinda new to this whole thing and just want to make sure I do it right.	To sign up for the research, please contact the Research Coordinator at [REDACTED] or at [REDACTED]. They will guide you through the specific process and next steps you need to follow.
	So, if I get picked for this study, does that mean I definitely have Alzheimer's, dementia, or some kind of brain issue? I'm just trying to figure out what it really means to be eligible.	No, being eligible to participate in the study does not mean that you have Alzheimer's, dementia, or any cognitive disorder. It's important to consult your physician for a proper diagnosis.
**Payment**	So, um, can you tell me what the compensation is for participating in this? I'm kinda curious about that.	Yes, you will be paid for participating in the study. All participants will receive the same amount for each assessment they complete, ensuring equal compensation.
	So, um, if I happen to finish just a portion of the work, will I still get compensated for that? Just curious about how that works!	If you only complete a portion of the work, you will still be compensated for the assessments you finished before leaving the study. However, you won't receive payment for any sessions that you do not complete after you decide to quit.
**Risks and Benefits**	Hey, could you let me know what kind of dangers or downsides might come with taking part in this research? I'm kinda curious because I've never done anything like this before and just want to make sure I'm aware of everything.	Participating in this research has minimal expected risks, but it's important to be aware of some mild potential physical and emotional risks. The main concerns are related to the MRI scan and the possibility of mental health or safety issues, in which case the study team may contact your primary care physician to ensure your well-being. Overall, the study is designed to minimize these risks.
	I'm curious, what are the advantages I might gain from participating in this research? How could being part of this study be useful for me?	While this research study is not intended to provide direct benefits to you individually, there are potential indirect advantages. Participating could engage you in regular cognitive activity, which is recommended for maintaining cognition and quality of life in older adults. Additionally, your involvement may contribute to research efforts that could lead to improved treatments or recommendations for you and others in the future. Many participants also find value in helping advance scientific knowledge and assisting others.

**Study Details**	Um, could you tell me where I might get my brain MRI done? I'm kinda new to all this and just want to make sure I know where to go. Thanks!	You will receive your MRI brain scans at the [REDACTED], located at [REDACTED]. A staff member will meet you outside to assist with parking and escort you into the [REDACTED].
	Um, could you tell me the duration of this research project? I'm kinda curious about how much time it will take since I'm considering getting involved.	The duration of the research project will be approximately 5 months. During this time, you'll have a series of appointments, including cognitive assessments and MRI brain scans, along with a 6-week cognitive training program involving up to three 1-hour sessions per week.
**Results**	am I gonna get to check out my own results after this?	Yes, you will be able to check out your own results after the study. Study coordinators will summarize your assessment scores and provide them to you on or after your last study visit. However, keep in mind that these results are not intended for diagnosis, and it's best to discuss any concerns with your regular doctor.
	I'm kinda curious, what kind of insights can I gain about my well-being from the results of this evaluation?	The assessment results will provide insights into your cognitive performance, but it's important to remember that they should not be considered a clinical evaluation or used for diagnosing any conditions. If you have questions or concerns about your results, discussing them with your regular doctor can help you gain a better understanding of your well-being. While the study is not designed to provide direct benefits to you, engaging in cognitive activities through the research may help maintain your cognitive health and quality of life.

Prompts

Rephrasing

The prompt for rephrasing the questions in the Q/A database:

“Rephrase the given question with no lexical overlap with the original.
Vary vocabulary, sentence structure, implicit vs explicit phrasing, etc.
Rephrase the question as if you are a human asking the same question.
  Since you are human, you will have a personality, diverse background, demographics, socioeconomics, you will come from certain regions in the US, etc. Ask the question from these perspectives without explicitly stating your perspective, who you are, or background.
Be creative.
Sometimes you can add a one or two sentences of context to your question (context about why you are asking or why you are interested in joining this study or even joining ANY research study).
Sometimes you can pretend that you've never used a chatbot before or that you've never joined a research study before.
  So maybe you don't know what you are doing, are nervous, make simple mistakes, get confused, etc.

You can also add misspellings, incorrect or no punctuation, different cases (lower, capital), etc. Be creative and vary your rephrasings

Also do not rephrase the question if it is already in the list of previous versions: {previous_versions}

Provide only rephrased question."

LLM as a Judge

"Evaluate the similarity between the two responses to the question below on a scale of 1 to 10:

Question: {question}
Ground Truth: {ground_truth}
RAG Response: {rag_response}

Provide only the number rating as your response."
